# Narrative Review of the Theoretical–Methodological Foundations of the TREINI Program

**DOI:** 10.3390/children11101181

**Published:** 2024-09-27

**Authors:** Renato Guimarães Loffi, Deisiane Oliveira Souto, Thalita Karla Flores Cruz, Arthur Felipe Barroso de Lima, Fabiana Rachel Martins Costa Rocha, Simone Rosa Barreto, Patrícia Aparecida Neves Santana, Amanda Aparecida Alves Cunha Nascimento, Vitor Geraldi Haase

**Affiliations:** 1 Institute of Neurodevelopment, Cognition, and Inclusive Education (INCEI), Ltd., Rua Carmélia Loffi 17, Justinópolis, Ribeirão das Neves 33900-730, MG, Brazil; 2 Rehabilitation Sciences Program, Department of Physiotherapy, Federal University of Minas Gerais, Belo Horizonte 31270-901, MG, Brazil; 3 Graduate Program in Neurosciences, Federal University of Minas Gerais, Belo Horizonte 31270-901, MG, Brazilvitorghaase@treinitec.com.br (V.G.H.); 4 Graduate Program in Psychology, Cognition and Behavior, Federal University of Minas Gerais, Belo Horizonte 31270-901, MG, Brazil; 5 Graduate Program in Speech-Language Sciences, Federal University of Minas Gerais, Belo Horizonte 31270-901, MG, Brazil

**Keywords:** cerebral palsy, intensive intervention, interdisciplinary program, family-centered practice, therapeutic suit

## Abstract

Scientific knowledge has advanced in the implementation of safe and beneficial interventions for children and adolescents with cerebral palsy (CP). Although the importance of interdisciplinary interventions that integrate all components of the International Classification of Functioning, Disability and Health (ICF) into family-centered practices is widely recognized, this approach is not yet widely adopted. Instead, many programs remain focused on isolated domains. This study presents the theoretical and methodological foundation of TREINI, an interdisciplinary and family-centered program developed for children and youth with CP and other neurodevelopmental disorders. TREINI incorporates intervention strategies that address all ICF domains. It is grounded in the biopsychosocial model of health and utilizes principles based on the best evidence in pediatric rehabilitation, including intensive training, task-oriented training, and a naturalistic learning environment. Unlike traditional rehabilitation approaches, the care provided by the TREINI program is delivered through an intensive and interdisciplinary approach, by a team working collaboratively in a single location. In addition to including evidence-based interventions, the TREINI program features two innovative components: the “City of Tomorrow”, a naturalistic learning environment, and the “TREINI Exoflex” therapeutic suit, specifically designed to address deficiencies in the body functions and structures of children with CP and other neurodevelopmental disorders. This program has been carefully designed to support the process of neurological re-education and rehabilitation for children and adolescents with neuropsychomotor developmental delays.

## 1. Introduction

Scientific knowledge has significantly progressed in terms of developing interventions and approaches that are safe and beneficial for children and youth with disabilities [1]. The implementation of the International Classification of Functioning, Disability and Health (ICF) by the World Health Organization in 2001 brought about significant changes in pediatric rehabilitation [2]. The ICF has led to a shift from exclusively focusing on impairments in body structures and functions to a more holistic perspective, recognizing the importance of interventions that also consider components of activity and participation. Additionally, the importance of providing children with opportunities to learn to move in natural and real-life environments while engaged in meaningful activities is acknowledged [3]. This approach is based on the premise that motor and functional development is optimized when children are exposed to real and challenging contexts that reflect the demands and complexities of everyday life [4].

The change in the focus of the intervention, emphasizing full participation in the community, integrates clinical reasoning and decision-making, and takes into account the individual preferences of children, youth, and their families. The current scientific literature strongly supports the adoption of family-centered interventions, considered the gold standard in pediatric rehabilitation [5]. In this model, both the child and the family are fully involved in all stages of the decision-making process, expressing their priorities and desires to healthcare professionals who work closely with the families [5]. Client-centered goals, the direct practice of goals by the individual, and the adaptation of tasks and the environment to meet the individual’s needs are aligned with the personal and environmental factors of the ICF.

Despite the recognized importance of interventions that consider all ICF domains aligned with family-centered practices, the majority of programs are still focused on isolated domains. The systematic review by Novak et al. [6] evaluated the levels of evidence of 182 interventions aimed at children with cerebral palsy (CP). Among these interventions, 383 outcomes were identified; approximately 62% were directed at the level of body structure and function, 13% at the level of activity, 3% at the level of participation, 3% at the level of environmental factors, and less than 1% were directed at the level of personal factors. The review by Novak et al. [6] also showed that only 15% of interventions targeted a combined level of body structures and activities, and 3% were focused on a combined level of activities and participation. Furthermore, of the 182 interventions analyzed, only 14% presented robust evidence of efficacy, confirming the need for more research in this area.

Thus, despite substantial advances in pediatric rehabilitation, there is still a vast field for progress. It is imperative to intensify our efforts in providing effective interventions that address the different ICF domains. Moreover, intervention programs should be proposed in an interdisciplinary manner, given that the complex needs of children with disabilities and their families cannot be fully met without the interaction and communication of an interdisciplinary team [7]. Despite the recognized importance of interdisciplinarity, challenges still exist for its faithful implementation. Collaboration among various healthcare professionals allows for a more comprehensive and effective approach, improving outcomes for children and their families. Thus, investing in interdisciplinary practices and promoting communication and coordination among team members are essential steps to achieving high-quality rehabilitation.

The current study aims to present a narrative review of the theoretical and methodological foundations underpinning the TREINI Program, (from Portuguese Treinamento em Reabilitação Neurológica Intensiva, or Intensive Neurological Rehabilitation Training), an interdisciplinary, comprehensive, and intensive intervention aimed at neurodevelopmental disorders such as CP, Down syndrome, and myelomeningocele. The program is based on scientifically proven neurological interdisciplinary intervention techniques and practices combined with the application of technologies such as the City of Tomorrow and the TREINI Exoflex therapeutic suit.

The TREINI program presents a distinct approach compared to other intensive interventions, such as Constraint-Induced Movement Therapy (CIMT) and Hand–Arm Bimanual Intensive Training (HABIT). While the latter are recognized for their intensive nature, their application is limited to the rehabilitation of upper limbs. In contrast, TREINI adopts a more comprehensive and multidimensional perspective. A crucial aspect that differentiates TREINI is its emphasis on interdisciplinarity, which is not addressed in CIMT and IBT. The TREINI program integrates knowledge and practices from various health areas, such as physiotherapy, occupational therapy, speech therapy, and psychology, providing a holistic approach to the rehabilitation process. This interdisciplinary integration allows for a more complete assessment and intervention, addressing not only motor aspects but also cognitive, emotional, and social aspects of the patient. This global approach is aligned with the most recent recommendations of the World Health Organization as to rehabilitation [2], which emphasize the importance of comprehensive and patient-centered interventions. TREINI’s interdisciplinarity also favors the transfer of skills to real-life contexts, an aspect often neglected in more focused approaches [3]. Recent studies demonstrate that interdisciplinary interventions have greater potential to improve patients’ functionality and quality of life, compared to unidisciplinary approaches [7]. TREINI stands out for its intensive, interdisciplinary, and comprehensive approach, overcoming the limitations of more restricted interventions such as CIMT and HABIT, and aligning itself with contemporary evidence-based rehabilitation guidelines.

This paper emphasizes the distinctive aspects of the TREINI program, thoroughly examining the theoretical foundations and evidence supporting specific interventions, with a focus on the motor interventions that constitute the program. Although the intervention program is applicable to other health conditions, the current study will focus on children with CP, an area in which the highest concentration of research is in pediatric rehabilitation. This article is structured into the following sections: Definition and Clinical Epidemiology of CP; Biopsychosocial Impacts of CP; Interventions for CP; Characterization of the TREINI Program (including theoretical foundations); City of Tomorrow: A Naturalistic Environment; Therapeutic Suit Based on Myofascial Tracks (TREINI Exoflex); TREINI Program Evaluation Strategy; and Final Considerations.

## 2. Definition and Clinical Epidemiology of CP

CP is defined as a group of permanent disorders that affect the development of movement and posture, resulting in activity limitations and participation restrictions [8]. These disorders are attributed to non-progressive lesions or abnormalities that occur in the developing brain. Risk factors for CP range from the preconception period to the early years of life [8]. Identified factors include genetic variants, congenital anomalies, prematurity, intrauterine growth restriction, infections, hypoxic–ischemic events, cerebrovascular insults, and both accidental and non-accidental brain injuries [9]. These factors can interact in complex causal pathways, contributing to the etiology of CP.

The clinical presentation and functional characteristics of CP are varied and commonly accompanied by cognitive, sensory, epileptic, musculoskeletal, and other alterations [8]. CP encompasses a wide range of children and adolescents with diverse clinical profiles. To systematize the categorization of individuals with cerebral palsy, different classification systems have been developed based on clinical characteristics and neurological profiles. The European classification of CP segments the condition into four main types: spastic, dyskinetic, ataxic, and mixed. Spastic cerebral palsy, the most prevalent, is associated with lesions in the pyramidal system and is characterized by increased muscle tone and rigid movements. It can be topographically subdivided into spastic hemiplegia, diplegia, and quadriplegia, depending on the limbs affected. Dyskinetic cerebral palsy, related to lesions in the basal ganglia and their connections, manifests as involuntary movements such as dystonia, athetosis, and chorea. The ataxic form results from lesions in the cerebellum and its connections, causing balance and coordination problems. Mixed cerebral palsy involves lesions in multiple systems, often combining spastic and dyskinetic symptoms. Additionally, the classification considers the severity of the condition, which can impact independence and mobility, and categorizes individuals as tetraplegic, diplegic, hemiplegic, or unilateral and bilateral [10].

Other forms of classification for CP are also available; some have an emphasis on functionality, specifically addressing mobility, manual dexterity, and communication. These systems include the Gross Motor Function Classification System (GMFCS) [11], Manual Ability Classification System (MACS) [12], Communication Function Classification System (CFCS) [13], Eating and Drinking Ability Classification System (EDACS) [13] and Visual Function Classification System (VFCS) [13], among which the GMFCS is recognized as the most significant classification tool for children with CP [14]. This system is widely used by healthcare professionals to predict mobility and locomotion capabilities, guide the planning of therapeutic interventions, and determine the prescription of assistive technologies and mobility aid devices.

CP is the leading cause of motor disability in childhood, with variations in incidence between different countries [15]. In high-income countries, the prevalence is approximately 1.6 per 1000 live births. In contrast, in low- and middle-income countries, the prevalence of CP is still uncertain, but it is believed that the rates are higher due to the high rate of infectious diseases and inadequate prenatal and/or perinatal care [15]. Evidence from developed countries indicates that approximately one in three children with CP is unable to walk, one in four has speech difficulties, one in four suffers from epilepsy, and one in twenty-five has a hearing impairment [16,17]. Studies indicate reductions in the prevalence of functional levels classified as ‘moderate to severe’ among children with CP in Australia over the past few decades [18]. In contrast, in low- and middle-income countries, children with CP present more severe physical limitations and a significantly higher incidence of comorbidities compared to children in developed countries [19].

## 3. Biopsychosocial Impacts of CP

The ICF is based on the biopsychosocial model of health [2]. This model provides a comprehensive conceptual framework for understanding the functionality process of individuals, integrating biomedical, psychological, and social factors [2]. According to this approach, functionality is an overarching term that includes all body structures and functions, activities, and participation. Similarly, disability is a concept that encompasses the presence of impairments in body structures and functions, activity limitations, or participation restrictions. Functionality and disability are seen as the result of the dynamic interaction between an individual’s health condition and contextual factors (environmental and personal). Children with CP exhibit impairments in all functionality domains of the ICF, a finding which we will address in detail throughout this section.

### 3.1. Impairments in Body Structures and Functions

Muscle weakness is a primary characteristic in all children with CP. In addition to central nervous system injury, this weakness can be attributed to various factors, including altered muscle development, the presence of spasticity, and muscle disuse [20,21]. Structural, mechanical, and biological alterations in the muscles of these children are well documented [20,22]. Among the most frequent changes reported are modifications in the proportion of type I and type II muscle fibers, with atrophy of type II fibers; reduction in muscle fiber size; disorganization of sarcomeres and reduction in the number and function of mitochondria; changes in contractile proteins and energy metabolism; increased connective tissue; and alterations in the neuromuscular junction [23,24]. All these modifications directly impact the ability to generate muscle strength. It is estimated that the muscle strength of children with CP is about 50% lower compared to their typically developing peers [25].

Factors such as force generation, endurance, effort, and biomechanical changes, including variation in the muscle length–tension curve, biomechanical degrees of freedom, sensory inputs, and postural control, are crucial in movement planning in children with CP [26]. The reduction in active muscle strength, in addition to the loss of corticospinal tract projections, is linked to the intrinsic properties of muscle and connective tissues, such as decreased muscle size, replacement by fat, reduction in serial sarcomeres, and loss of titin, affecting force production [27,28]. Anomalies in sarcomerogenesis, decreased numbers of satellite cells, increased amounts of connective tissue and fat, elastic microfibrils, and ribosomal dysfunction are also observed in spastic muscles [26,28,29]. Disproportionate muscle growth relative to bone and increased intramuscular connective tissue contribute to contractures, while biomechanical changes generate abnormal forces on growing bones, resulting in deformities such as femoral neck anteversion and congenital hip dislocation [30,31].

Spasticity, resulting from upper motor neuron injury, is the main disorder in the majority of children with CP, affecting around 70% to 90% [32]. Spasticity causes excessive and uncoordinated muscle activation, which can lead to increased muscle hypertonia. Children with hypertonia exhibit increased passive muscle stiffness, changes in muscle composition and connective tissue, and a reduction in muscle fiber length. These changes limit movement and muscle stretching, hindering the longitudinal growth of muscles and their functions [33]. Spasticity can lead to the development of contractures and bone deformities, such as scoliosis and hip dysplasia, as well as causing muscle and joint pain [33].

Children with CP often exhibit changes in postural control [34]. The definition of postural control is the ability to manage the position of the body in space, with the aim of achieving orientation and stability [34]. Dysfunction in postural control in children with CP results from primary brain injuries, which lead to deficits in postural networks [35]. Compared to typical children, those with CP exhibit deficits in anticipatory and reactive postural adjustments, as well as in the sensory and musculoskeletal components of postural control [35]. These deficits contribute to limitations in gross motor skills that require balance and gait. It is important to note that deficits in postural control may persist even after the child has acquired the ability to stand and walk independently [36].

Children with CP often exhibit reduced balance and tend to disproportionately rely on visual information to maintain posture and position their limbs during gait, suggesting proprioceptive impairment. Proprioceptive deficiencies occur mainly due to injuries in the central nervous system, where proprioceptive stimuli generated by muscle spindle, Golgi tendon organ, and joint and cutaneous receptors are affected within the cortex [37]. Additionally, spasticity, which is present in most children with CP, compromises proprioception by impairing the relationship between the muscle and the joint [37]. Proprioceptive disorders in individuals with CP can negatively impact performance in various activities, including activities of daily living.

Secondary body impairments, such as scoliosis and hip dislocation, are frequently observed in children with CP, especially in those with greater functional impairment [38]. Scoliosis is the most prevalent alteration and is closely associated with pelvic obliquity and hip misalignment. Hip instability is a common dysfunction in children with CP and can progress to subluxation and dislocation. Factors such as increased femoral neck anteversion, changes in femoral remodeling due to the lack of independent walking acquisition, and abnormal weight-bearing patterns, common in children with CP at levels III to V of the GMFCS, are also associated with hip dislocation [30].

Although this topic has emphasized neuromusculoskeletal functions related to movement, it is crucial to recognize that children with CP may present impairments in various other body structures and functions. Children with CP are at a higher risk of developing cognitive impairments [39]. The extent of these cognitive impairments varies both between and within the spastic, dyskinetic, and ataxic subtypes. In the subgroup with spastic quadriplegia, it is estimated that between 90% and 100% of children have an IQ below 70 [40]. Brain disorders and gray-matter injuries are associated with higher rates of intellectual disability [41]. Overall, children with CP are at risk of intellectual disability of varying intensities. A population-based study involving 1141 children with CP reported that 45% of them presented intellectual disabilities [41].

Children with CP may also exhibit deficits in executive functions [42]. Functional neuroimaging research indicates that the prefrontal cortex, along with the white-matter tracts connecting the prefrontal and posterior regions of the brain, plays a crucial role in the development of executive functioning skills [43]. Thus, children with CP with injuries in these areas are more likely to experience difficulties related to executive functions. There is evidence that children and adolescents with CP demonstrate significant deficits in various executive functions compared to their typically developing peers, including difficulties in inhibitory control, working memory, visual and auditory attention, cognitive flexibility, planning, and information processing [44,45]. In the study conducted by Bottcher et al. [45], attention and executive function were analyzed in children with CP using standardized neuropsychological measures. The findings revealed deficits in these functions, offering a potential explanation for the social and learning difficulties observed in this group.

Speech disorders are highly prevalent among children diagnosed with CP, affecting approximately 60% of this population [46]. Among the most frequently observed disorders is dysarthria, which is present in about 78% of verbal children with CP, along with phonological disorders (43%) and childhood apraxia of speech (17%) [46]. Speech disorders are mostly due to problems in motor control of the muscles involved in speech production. The incidence of dysphagia is significantly elevated in children diagnosed with CP, especially in cases with more severe neurological deficits. Oropharyngeal dysfunction, which impairs swallowing ability, affects up to 90% of these children. Due to this high prevalence of dysphagia, many children with CP require alternative feeding methods, such as the use of feeding tubes, to ensure adequate nutrition and avoid complications associated with malnutrition [47]. Changes in oral motor function can create disabilities in each stage of the swallowing process, resulting in malnutrition, dehydration, aspiration, and pneumonia [48]. Swallowing changes in children with CP have been associated with a decline in gross motor function [47,49].

Changes in sensory functions and pain [50] and dysfunctions in the cardiovascular and respiratory systems [51], as well as visual [52] and auditory deficits [53], may also be present in CP.

### 3.2. Activity Limitations

The limitations in functional activities observed in children with CP are influenced by a variety of factors; however, these limitations can be partially anticipated based on the clinical classification and type of CP. Children with CP classified at level I of the GMFCS and MACS tend to present less significant restrictions in their daily activities [54]. Nevertheless, they still face various functional limitations. Among these limitations, difficulties in functional mobility are prominent, including the inability to change and maintain postures, perform transfers, walk, and move in different environments [55]. Difficulties in self-care activities and activities of daily living (ADLs) are also common in children with CP [56], particularly, feeding, personal hygiene, and dressing. Additionally, limitations in handling, grasping, and fine pinching are frequently observed; these reduce the ability to carry, handle, or move objects with the upper limbs [57]. Communication ability is also significantly affected in children with CP; it is estimated that approximately 50% of these children have some type of speech problem, and approximately 25% are classified as non-speaking [58]. Communication changes in this population are complex and multifaceted, affecting verbal, non-verbal, receptive, written, and alternative communication.

Children with CP often face limitations in academic progress due to a variety of learning difficulties [59]. Difficulties in acquiring skills in reading, writing, spelling, and mathematics are frequently observed in children with CP, with prevalences ranging from 30% to 70% [60]. These difficulties are influenced by factors such as low cognitive ability, attention and behavior problems, and psychosocial and motivational challenges, as well as impaired language skills. Specifically, approximately 60% of these children face communication-related challenges, including dysarthria and difficulties in speech and language, which can affect their daily interactions and school performance [42]. Additionally, approximately 30% of school-aged children with CP exhibit behavioral difficulties, such as problems relating to peers, emotional symptoms, and hyperactivity [61]. These factors not only interfere with the ability to concentrate and participate in school activities [60], but also significantly influence engagement and persistence in academic tasks.

### 3.3. Restrictions on Participation

Participation restrictions are also less significant in children with better levels of gross motor function and manual ability. There is consistent evidence of a significant association between the functional classification of children and adolescents with CP and the restriction in their participation in activities [62]. Children with CP face restrictions in various contexts, including home, school, leisure, recreation, and participation in sports activities [63]. Individuals with CP are more likely to experience low levels of participation in leisure-related physical activities compared to their typically developing peers [64]. Restrictions on participation in physical and sports activities are found not only due to impairments in body structure and function and limitations in the physical, cognitive, psychological, and social domains of physical literacy [65], but also due to contextual barriers at the personal and environmental levels of the ICF. The study by Souto et al. [63] found low levels of participation in recreation and leisure activities in children with CP, with an average participation frequency of two times in the previous 4 months. Children with CP often face restrictions in school participation due to a combination of physical, cognitive, emotional, and social factors. These restrictions can contribute to school dropout. A recent study conducted by Alves et al. [66] revealed that 22% of children with CP are not enrolled in educational institutions, while 10.4% are enrolled in special education schools.

### 3.4. Contextual Barriers and Facilitators

The functionality and quality of life of individuals with CP are profoundly influenced by a complex interaction of contextual factors. Access to physical spaces in different contexts (home, school, and community), along with family and school support, the use of assistive technologies, availability of programs, community transportation means, and socioeconomic level, play a crucial role in the functionality of children with CP [67,68]. These factors can act as barriers or facilitators, positively or negatively influencing the development and participation of these children in daily activities [68]. Among the barriers are inaccessible infrastructures, such as the absence of ramps and adapted bathrooms, lack of assistive technology, social stigma, and discrimination, as well as financial constraints that limit access to medical care and therapies [69]. On the other hand, facilitators include the presence of an accessible infrastructure, such as ramps and elevators, the availability of modern assistive devices, supportive attitudes from family and community, inclusive education policies, financial assistance programs, and inclusive employment opportunities [68].

Studies highlight the essential role of the family in the participation of children and adolescents [67,68,69,70]. One of the main barriers to participation in activities is the negative attitudes and behaviors of parents/caregivers and others. Similarly, positive attitudes and behaviors acted as facilitators for participation. A family environment that provides support, appropriate stimulation, and encouragement significantly increases the chances of the child reaching their full potential. Conversely, the absence of family support, whether due to stress, lack of knowledge, or limited resources, can compromise the child’s progress and social inclusion [71].

The school environment often presents multiple barriers that impact the daily lives of children with CP and other disabilities. Children with CP frequently present special needs in motor, cognitive, and learning areas, directly influencing their educational process and overall school experience [72]. School barriers encompass physical aspects, such as the lack of structural adaptations that facilitate mobility and access to school spaces; aspects related to the adaptation of pedagogical activities, both inside and outside the classroom; and social aspects, including the absence of policies that promote diversity and social inclusion. These barriers can compromise the development of these children, hindering their school-related and social inclusion, resulting in learning gaps, difficulties accessing spaces, and challenges in socialization.

## 4. Interventions for CP

In the past ten years, advances in high-quality research on CP have significantly driven the development of safer and more effective interventions for children with this condition. The systematic review conducted by Novak et al. in 2013 identified 64 distinct interventions available for the treatment of CP [73]. An update of this review in 2020 revealed a substantial increase, with 182 documented interventions, representing an increase of 118 interventions over a seven-year period [6]. This exponential growth reflects the ongoing effort of the scientific community to enhance therapeutic approaches and improve outcomes for these children. However, the rapid expansion of the evidence base has made it difficult for healthcare professionals to stay updated, and it is challenging for families to know how to best help their children. Unfortunately, outdated clinical care is regularly being provided to children with CP [16]; it is believed that approximately 10% to 40% of children with CP do not receive interventions with proven efficacy, and another 20% receive harmful or ineffective interventions [16]. Consistent with this statement, of the 182 interventions reviewed by Novak et al. [6], approximately 86% still require further studies to confirm their efficacy.

Interventions aimed at managing CP range from preventing its occurrence to reducing spasticity and improving function. In the realm of preventive measures for CP, significant progress has been made, leading to a 30% decrease in incidence rates in certain high-income nations, where the current prevalence stands at 1.4 per 1000 live births. Preventive strategies, including the antenatal use of magnesium sulfate and corticosteroids, have demonstrated considerable effectiveness in reducing the risk of CP [74]. Furthermore, prophylactic caffeine and therapeutic hypothermia have proven to be effective neuroprotective strategies for preterm and term infants, respectively. Within regenerative medicine, therapies like erythropoietin and umbilical cord blood are under investigation as possible neuro-regenerative treatments [75]. However, the implementation of these interventions faces challenges due to the lack of adequate legislation allowing access to treatments such as the use of umbilical cord blood as a therapeutic measure.

Although pharmacological agents are effective in reducing spasticity, there is less robust evidence on improvements in other aspects, such as functional mobility [6]. Pharmacological agents like botulinum toxin, intrathecal baclofen, diazepam, and selective dorsal rhizotomy are effective in reducing spasticity, while dantrolene and tizanidine are probably effective [73]. Local injections of alcohol and phenol are also capable of reducing spasticity, though they may come with side effects. Combining multiple interventions might offer additional benefits; for instance, pharmacological agents that decrease spasticity can aid in movement learning, while strength training can enhance muscle strength and endurance [76].

Some of the most effective motor interventions for children with CP include Goal-Directed Training [77], Task-Specific Training [78], Constraint-Induced Movement Therapy (CIMT) [79,80], and Hand–Arm Bimanual Intensive Training (HABIT) [81]. Additionally, home programs, treadmill gait training, mobility training, hippotherapy, environmental enrichment, strength training, and functional chewing training are also widely recognized for their effectiveness [6]. These interventions share fundamental characteristics, such as the practice of real-life tasks and activities, the use of self-generated active movements, and high practice intensity. The aim of these practices is directly aligned with goals set by the child, thus promoting active and meaningful engagement in the therapeutic process.

Although nearly half of children with cerebral palsy (46%) present concurrent intellectual disabilities, few interventions have been proposed to address the cognitive impairments in this population. Adapted literacy interventions using communication devices have been shown to be effective [82]. Additionally, infants who received the GAME intervention (motor training, environmental enrichment, and coaching) showed better cognition by their first year of life [83]. Another innovation is the CO-OP intervention (Cognitive Orientation to Daily Occupational Performance), in which children set goals and discover strategies to achieve them, practicing real-life tasks at high intensity [84]. Studies suggest that CO-OP improves function at a low cost and with large effect-sizes.

In addition to the lack of robust evidence of efficacy for most interventions, several issues hinder the clinical progression of children with CP. Firstly, one persistent problem in intervention programs is the difficulty in promoting the generalization of gains achieved in clinical settings to real-life environments. Most current rehabilitation programs are conducted in clinics and offices, and the improvements made in these contexts do not always transfer to the children’s everyday environments. Secondly, the complex needs of children and adolescents with disabilities, as well as their families, require the interaction and communication of an interdisciplinary health team. However, interventions provided by interdisciplinary teams are rare [85]. Thirdly, interventions are often offered in isolation and in different locations, making the treatment process even more challenging for families. Finally, interventions tend to focus on specific domains rather than considering all components of functionality and disability. Efforts are needed to promote interdisciplinary interventions in a single, enriched environment that considers all functionality domains according to the ICF.

Evidence on the treatment of speech motor disorders in children with CP shows that different approaches can improve intelligibility, articulation accuracy, and speech clarity, with variations in effectiveness depending on the intensity, frequency, and specificity of the interventions [86,87]. Motor learning principles are crucial for success, facilitating neuroplasticity and the development of speech motor skills [88]. Interventions follow a practice hierarchy, starting with isolated sounds and advancing to complete phrases, with adjustments made to complexity in order to promote adaptation and learning [89]. Augmentative and Alternative Communication (AAC) is also highly beneficial, improving communication in terms of vocabulary, auditory comprehension, and oral expression, as well as contributing to literacy, participation in daily activities, reduced frustration, and increases in quality of life and self-determination [90]. Immediate implementation of AAC is recommended for children with severe language deficits, as it supports communicative development [91]. In addition to direct interventions, training communication partners, such as parents and caregivers, is crucial for creating a supportive communication environment.

Findings on the treatment of dysphagia in children with CP highlight that the approaches with the most evidence in the literature include the combination of electrical stimulation with oral sensorimotor therapy, Functional Feeding Training (FFT), a multidisciplinary intervention, and caregiver guidance for the safe provision of food [6,92]. The combination of electrical stimulation with oral sensorimotor therapy improves lip closure function and the ability to swallow without anterior food escape, demonstrating additional benefits compared to oral sensorimotor therapy alone [92]. FFT, which uses direct and indirect interventions with sensory stimulation, is particularly effective in improving chewing and reducing tongue thrust and drooling. These techniques are well-founded and effective in clinical practice. Personalizing treatment, such as adapting food consistency to the child’s needs and guiding caregivers on feeding management strategies are essential for treatment efficacy. Interdisciplinary work is crucial to address pediatric dysphagia and promote effective and integrated rehabilitation [6,92].

Given that children with CP frequently face psychological challenges, including difficulties in social interaction [93], it is essential to implement interdisciplinary interventions that incorporate psychological components. These interventions should focus on enhancing social interaction skills and promoting literacy and numeracy [93]. This approach contributes to increased levels of learning in math, reading, and writing for the children involved. Additionally, an essential part of this process is the active participation of the family through Parent Guidance or Training, which can contribute both to extending the intervention to the home environment and to empowering caregivers to handle the challenges involved in caring for children with CP, especially when behavioral issues arise [94]. Parental involvement in the therapeutic process contributes not only to optimizing intervention outcomes, but also to improving the family’s quality of life and the interaction between parents and children

## 5. Theoretical Foundations of the TREINI Program

The complexity and severity of the biopsychosocial impacts associated with CP justify the need for integrative and intensive intervention programs, initiated as early as possible. These programs should be provided by a well-trained and integrated interdisciplinary team [95], operating in a single location and in close collaboration with the family, to achieve common goals [5]. These are the principles of the TREINI program, which is not limited to a single intervention method but constitutes an integrated and interdisciplinary program that incorporates multiple intervention strategies. This program aims to address as many impact levels as possible, working according to the ICF Functionality model and its complex interactions [2].

The TREINI program is an interdisciplinary, family-centered intervention specifically developed for children and young people with CP (and other neurodevelopmental disorders, such as Down syndrome and myelomeningocele). This program is designed to support the process of neurological re-education and rehabilitation for children and adolescents with neuropsychomotor developmental delays. Grounded in the biopsychosocial health model, TREINI employs principles based on the best evidence in pediatric rehabilitation, including intensive training, task-oriented training, and environmental enrichment. Unlike traditional rehabilitation approaches, the care provided by the TREINI program is delivered through an intensive and interdisciplinary approach, by a single team working collaboratively in one location. TREINI incorporates intervention strategies that address impairments in all ICF domains. The intensive approach of TREINI is based on the assumption that significant outcomes at the level of body structure and function, such as tissue remodeling and activity-dependent neuroplasticity, require intense and prolonged interventions. The main features of the TREINI program, its theoretical–methodological foundation, and the respective references, are described in Table 1.

The TREINI program applies the biopsychosocial model in an integrated manner, addressing the needs of children with neuropsychomotor dysfunctions holistically. This model considers the fact that health and well-being are influenced by a complex interaction of biological, psychological, and social factors. The principles of the biopsychosocial model guide the development of personalized interventions in the TREINI program, ensuring that each child receives a treatment plan tailored to their unique needs. The detailed initial assessment considers biological, psychological, and social aspects, allowing the interdisciplinary team to develop strategies that maximize each child’s rehabilitation potential. This personalized approach not only improves clinical outcomes but also increases family satisfaction with the therapeutic process. Biologically, the focus is on improving physical and neurological function using advanced technologies, such as the TREINI Exoflex therapeutic suit. Psychologically, the program promotes motivation, self-confidence, and emotional well-being, including behavioral therapies and emotional support. Socially, it emphasizes collaboration between the family and healthcare professionals, ensuring that therapeutic goals meet the family’s needs and encouraging the children’s social interaction.

In the TREINI program, the intensive approach is implemented in a naturalistic environment called the “City of Tomorrow”, and by using a flexible therapeutic suit, the “TREINI Exoflex”. These components of the TREINI program will be described in depth in the next section.

**Table 1:** Characteristics and theoretical foundation of TREINI.

Component	Description	References
Family-Centered Practice	TREINI is based on Family-Centered Practice, which recognizes the relevance of family involvement in the process of recovery and child development. This approach promotes active collaboration between healthcare professionals, the child, and their family, ensuring that the family’s needs, values, and preferences are integrated into the planning and implementation of treatment. The importance of this practice lies in creating an environment of support and trust, improving adherence to treatment and enhancing therapeutic results.	Myrhaug, Jahnsen and Østensjø, 2016 [96] King and Chiarello, 2014 [97] King et al., 2004 [98]
Family–professional collaboration	Family–professional collaboration is supported by the TRIENI program by facilitating an in-depth understanding of the needs and priorities of the child and family, allowing for the personalization of treatment plans. This effective collaboration improves communication, strengthens mutual trust, and increases satisfaction with the services provided. The family’s active participation in the therapeutic process is essential for the continuity of care at home, which is crucial for the child’s continued evolution. Furthermore, the collaborative approach empowers parents, increasing their skills and confidence in relation to supporting their children’s development, resulting in better functional and emotional outcomes for the child and the family as a whole.	An and Palisano, 2014 [5] King and Chiarello, 2014 [97] Myrhaug, Jahnsen, and Østensjø, 2016 [96]
Evidence-based interdisciplinary practice	In the TREINI program, the interdisciplinary team is crucial to providing holistic and effective treatment. Professionals such as physical therapists, occupational therapists, speech therapists, and psychologists collaborate to create a comprehensive and personalized treatment plan. Each specialist addresses different patient needs, from mobility and motor coordination to communication and cognitive skills. Coordination between team members ensures integrated and complementary interventions, maximizing therapeutic results.	Andrade et al., 2012 [99] Das and Ganesh, 2019 [100] Hanson et al., 2024 [101] Rahlin and Rheault, 2024 [95]
Biopsychosocial approach	TREINI employs interventions that cover all domains of the International Classification of Functioning, Disability and Health (ICF), ensuring a holistic and comprehensive approach to patient care and rehabilitation.	Chen et al., 2014 [102] Andrade et al., 2012 [99]
Naturalistic learning environment	The TREINI program is based on the assumption that interventions carried out in the child’s living environments or in similar contexts promote greater gains and have a greater capacity to generalize these results.	Morgan, Novak, and Badawi, 2013 [103] Rostami and Malamiri, 2012 [104] Dunst et al., 2001 [105]
Intensive and prolonged training	The TRIENI program assumes that significant outcomes in terms of body structure and function, and involving tissue remodeling and activity-dependent neuroplasticity, require intense and prolonged interventions. The implementation of intensive programs has been increasingly recommended. For each functional goal, an intervention time of 15 to 25 h is required, depending on the complexity of the goal. TREINI recommends conducting an intensive interdisciplinary intervention, with a workload ranging from 40 to 80 h per month, according to the specific needs of the child.	Bleyenheuft et al., 2015 [106] Figueiredo et al., 2020 [107] Jackman et al., 2021 [108]
Best practices in pediatric rehabilitation	The TREINI program is aligned with the most recent scientific evidence, incorporating essential elements to promote functionality in individuals with CP. These elements include goal-directed practice of real-life tasks, active movements self-initiated by the child, application of motor learning principles, creation of an enriched environment, and family participation and involvement in therapeutic planning.	Bak and Lee, 2021 [109] Zai et al., 2022 [110] Novak et al., 2020 [6] Novak et al., 2014 [16] Khamis et al., 2020 [92] Korkalainen et al., 2023 [88] Whittingham et al., 2013 [94]
Supervision and continued education	The TREINI program provides online supervision for professionals, in addition to offering continuing education through regularly held courses and conferences.	Snowdon et al., 2016 [111] Coughtrey et al., 2024 [112]
Care management through app	TREINI uses a mobile application, TREINI+, designed to efficiently manage interdisciplinary healthcare. The TREINI+ application facilitates the implementation of home programs by providing a tool for therapists to guide families in integrating clinical activities into the home environment, and can be expanded to other living contexts, such as schools, parks, and squares. Furthermore, it allows the family to insert videos or photos of the child or young person carrying out activities in their natural environments, enabling the interdisciplinary team to observe the environment and behavior of the child or young person in activities that are meaningful to them and their family.	Fenning et al., 2022 [113] Zamin et al., 2019 [114]

## 6. City of Tomorrow

**Figure 1:**
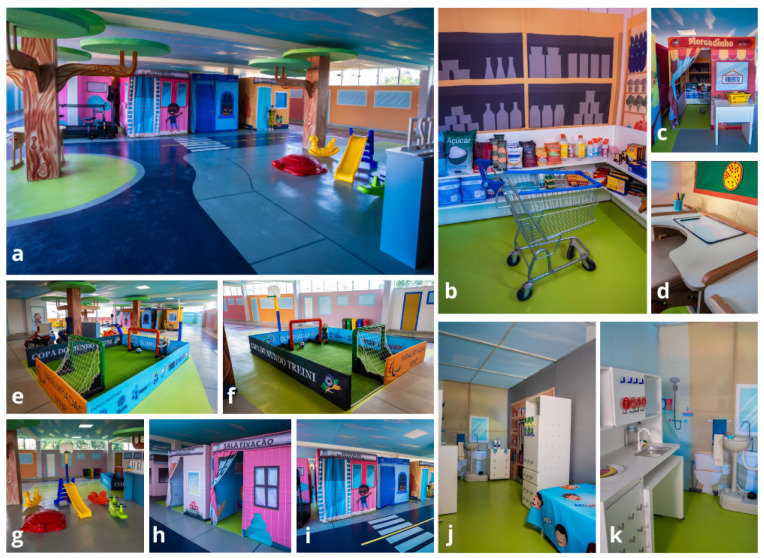
City of Tomorrow units. Image (**a**) shows the entrance hall of the City of Tomorrow. Images (**b**,**c**) illustrate the Market unit, where participants can engage in simulated shopping experiences. This unit is crucial for practicing daily living skills, enhancing cognitive functions, and promoting social interaction in a controlled and supportive environment. Image (**d**) presents part of the School unit, which replicates a classroom setting. This unit is essential for educational activities and cognitive rehabilitation, allowing participants to work on academic skills and social behaviors in a natural context. Images (**e**,**f**) show the Fitness Space unit, a space used to promote physical fitness. This unit supports the development of motor skills and encourages an active lifestyle. Images (**g**–**i**) provide external views of some units of the City of Tomorrow, highlighting the interconnectedness and accessibility of the different therapeutic spaces. These views emphasize the holistic approach of the environment, where various aspects of daily life are integrated into the rehabilitation process. Finally, images (**j**,**k**) illustrate the Home unit, a space designed to mimic a residential setting. This unit allows participants to practice essential daily tasks and self-care activities in a realistic yet controlled environment, fostering independence and confidence in their abilities.

In the TREINI program, interventions are administered through an intensive and interdisciplinary approach, by a team operating collaboratively in a single location called the “City of Tomorrow”. This immersive therapeutic environment is designed to enhance the engagement of children and adolescents, consisting of units known as naturalistic learning environments [115]. In these units, children have the opportunity to participate in meaningful activities for daily life in contexts that simulate their natural environment, such as home, school, and small grocery stores, among others. The “City of Tomorrow” is composed of playful and attractive spaces, specifically designed to increase the interest of children and adolescents in actively interacting with the environment, facilitating the execution of the proposed activities [115]. These spaces promote sensory stimulation, active movement, communication, and cognition. Additionally, the environment incorporates reinforcement systems based on Applied Behavior Analysis and uses structured cognitive learning models grounded in cognitive load theory, which supports the development of cognitive schemas. Figure 1 and Table 2 provide illustrations and details of the units of the City of Tomorrow.

## 7. Therapeutic Suit (TREINI Exoflex) Based on Myofascial Meridians and Tensegrity

Recent studies highlight the growing importance of activity and participation-centered interventions, which have led to reduced emphasis on approaches focused on body structure and function [6]. However, it is crucial to recognize that while promoting activity and participation is essential, it does not eliminate the need to manage and prevent damage to body structures and functions [3], especially in children with CP, who exhibit various musculoskeletal system alterations [8,26,28]. Interventions targeting impairments in body structures and functions are fundamental, not only to facilitate more effective activity performance and increased participation, but also to prevent future musculoskeletal complications that may limit the child’s participation in activities and compromise their quality of life.

**Figure 2:**
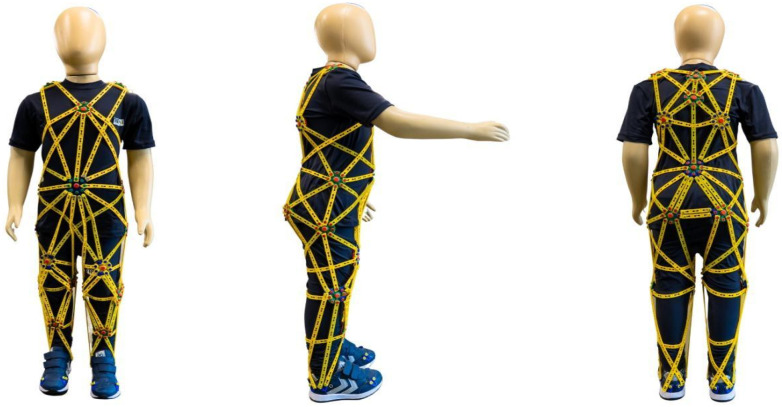
TREINI Exoflex therapeutic suit in anterior, lateral, and posterior views. The assembly of the suit is carried out by connecting the viscoelastic strips, or myofascial strips, to the fixation points, called nodes. Initially, a basic assembly is performed, after which modifications are made according to the postural correction needs of each child (determined through structural analysis).

In this context, this section presents the TREINI Exoflex therapeutic suit (Figure 2), which is designed to focus on body structures and functions, primarily addressing deficits in postural control, balance, and muscle strength in children with CP. These deficits are well-documented and were described in Section 2 of this manuscript, which discusses the biopsychosocial impacts of CP. Although other therapeutic suits have been developed for this purpose, the theoretical foundation and mechanism of action of the TREINI Exoflex suit differ significantly from these others. Initially, this section provides a literature review on the effects of therapeutic suits in children with CP. Subsequently, the theoretical foundation and specific mechanisms of action of the TREINI Exoflex will be addressed, highlighting its potential advantages and contributions to the clinical management of CP.

Maintenance of an adequate static posture and efficient dynamic control of movements are essential for the body to respond effectively to the demands imposed by daily activities [130]. Therapeutic suits can enhance the rehabilitation outcomes of children with developmental disorders by promoting posture and movement. There is currently a wide range of suits available. The first models were inspired by the “Penguin Suit”, an innovation from the Soviet space program of the 1960s [131]. This pioneering suit was designed to provide resistance to the movements of astronauts, facilitating tissue and biomechanical adaptation in microgravity environments. Its main objective was to mitigate the adverse effects of the absence of gravity, such as muscle atrophy and osteopenia, as well as to maintain the neuromuscular fitness of astronauts during prolonged missions in space. Subsequently, other suits were developed in association with intensive and specific treatment protocols for neurofunctional rehabilitation, such as the Adeli Suit, TheraSuit, and PediaSuit. These models incorporate hooks that anchor a system of elastic tubes individually fixed to provide traction between the trunk and pelvis and between the pelvis and lower limbs. The proposed mechanism of action used to explain the effects provided by these therapeutic suits is the continuous compression exerted by the suit’s elastic elements on the child’s musculoskeletal system [132].

The current literature does not seem to support the use of therapeutic suits in the rehabilitation of children with CP, due to a lack of robust evidence of their benefits [6]. A personal systematic search of the literature revealed four systematic reviews published between 2016 and 2019, as well as four clinical trials published between 2021 and 2024. The complete search strategy across different databases is detailed in Supplementary Material File S1. Table 3 and Table 4 present a summary of the studies found. The studies included at least 10 different types of therapeutic suits, and the sample included in the studies was quite heterogeneous, involving children, adolescents, and adults at different GMFCS levels. The methodological quality of the studies ranged from low to high. The results were mixed and, collectively, do not allow for definitive conclusions about the benefits of the suits in individuals with CP. It is possible that methodological flaws in the studies hinder the verification of the real benefits of therapeutic suits.

In addition to concerns related to the methodological quality of the studies, the proposed mechanisms of action of the available therapeutic suits have been widely questioned. Most therapeutic suits are based on the mechanical principles of resistance [131]. The mechanical model proposed by these suits is based on compressive forces aimed at structural stability. The principle of stability suggests that the better the alignment of body structures, the more stable the structure as a whole will be. This model indicates that different parts of the body have an ideal fitting form, such as the connection between the sacrum and the iliac wing or the head of the femur in the acetabular fossa. This fitting depends on friction forces, which promote adaptation and improve stability [133]. However, several limitations have been identified regarding the principle of stability based on shape and force [133,134]. The first limitation refers to the inability of joint surfaces to withstand compressive loads, which can lead to degenerative processes, even in younger children [133]. Another limitation is that the perfect fit occurs only in a specific position, not remaining stable across the various ranges of motion allowed by the joints [134]. This principle, which operates exclusively through compressive forces, restricts the diverse functional activities and movements performed by the human body [133,134].

Considering these constraints, the TREINI Exoflex therapeutic suit was developed. Given that compression-based models are insufficient to explain the stability of the musculoskeletal system, the TREINI Exoflex is partly founded on the principles of tensional integrity (or tensegrity) in the musculoskeletal system [134,135,136]. A tensegrity system is characterized by being intrinsically stable, and composed of compressive elements that are embedded in a continuous network of tensioned components [135]. Applying this concept to the musculoskeletal system, bones function as the discontinuous compressive elements, while soft tissues—including fascia, ligaments, muscles, and tendons—form the continuous network of tension elements that intrinsically connect the bones [135,136]. It is assumed that the organization of the musculoskeletal system aligns with tensegrity structures, with pre-stress present in the connective tissues of the musculoskeletal system.

Investigations into these continuous networks of tension elements have sought to understand how these connections occur and whether permanent anatomical connections exist. From this perspective, Myers [137] and Stecco et al. [138,139] describe models explaining eleven myofascial meridians, also known as “anatomy trains”, which describe lines of connection extending between tendons and muscles and are formed by tension elements (myofascial tissue), facilitating movement and providing stability to the body. According to Myers [137], myofascial meridians, from the perspective of tensegrity, are continuous bands along which tensile stresses are transmitted from one bony structure to another. These trains are directly involved in the organization of movement as well as the transmission of muscular force [137,138,139,140,141].

**Table 3:** Characteristics of the systematic reviews.

Study	Objective	Included Studies	Participant Characteristics	Interventions (Suits)	Intervention Intensity	Comparisons	Methodological Quality	Conclusion
Martins et al., 2015 [142]	Evaluate the effectiveness of the suit on the functionality of children with CP	4 RCTs published between 2006 and 2011	– Age: 3 to 13 years – Sample size: 20 to 36 (total of 110) – GMFCS: I to V	TheraSuit (1) AdeliSuit (2) Modified suit with emphasis on lower limbs (1)	– Minimum: 2 h/day (20 min of use), 3 weeks; – Maximum: 4 h/day, 5 times a week, 3 weeks	TheraSuit without elastic (1) Bobath (2) CT (1)	Low risk of bias on the PEDro Scale (score of 6.25)	Small improvement in gross motor function, with an effect size of 0.10
Almeida et al., 2017 [132]	Evaluate evidence on the effects of the suit on the impairments and functional limitations of children with CP.	13 studies published between 2000 and 2015: – 6 RCTs – 5 QE – 2 Case studies	– Age: 2 to 18 years – Sample size: 1 to 51 – GMFCS: I to IV	– Full Body Suit (2) – DEFO (2) – TheraTogs (2) – TheraSuit or AdeliSuit (6)	– Minimum: 50 min per day, once a week for 18 weeks; – Maximum: 10–12 h per day, 5 times a week, for 12 weeks	– Bobath (3) – CT (1) – Non-elastic suit (1) – No intervention (3) – Postural correction and gait training (1) – NR (4)	Quality varied from low to high as assessed by the Checklist for Measuring Study Quality	The DEFO and TheraTogs suits appear to improve postural alignment and gait performance in children with diplegic CP (evidence ranges from low to very low)
Wells, Marquez, and Wakely, 2017 [143]	Verify if suit therapy improves motor function in children with CP.	16 studies published between 1995 and 2016: – 5 RCTs – 11 Case studies	– Age: 15 months to 17 years – Sample size: 1 to 57 – GMFCS: I to V	– TheraTogs (2) – TheraSuit (3) – AdeliSuit (4) – UpSuit (1) – Lycra suit (4) – Dynamic Elastomeric Fabric Orthosis (1) – Various types of suits (1)	– Minimum: 1 h/day, 5 times a week, for 4 weeks; – Maximum: 12 h/day, 5 times a week, for 12 weeks	– Bobath (3) – Non-elastic suit (1) – No intervention (12)	Average 19.5 (out of 27) on the Downs and Black Scale; Variable quality	The use of the suits did not improve motor function.
Karadağ-Saygı, and Giray, 2019 [144]	Evaluate the effectiveness of the suit therapy for CP.	29 studies published between 1995 and 2018: – 10 RCTs – 8 QE – 11 Case studies or reports.	– Age: 3 to 14 – Sample size: 16 to 51 – GMFCS: I to V	– TheraSuit (5) – AdeliSuit (5) – TheraTogs (2) – PediaSuit (1) – UpSuit (1) – Lycra Suit or arm splint (5) – Different splints (1) – Pressure suit (3) – NR(6)	– Minimum: 2 h/day, 5 times a week for 4 weeks; – Maximum: 6 h/day, 5 times a week for 3 months	– Bobath (3) – CT (3) – Non-elastic suit (1) – Postural correction (1) – TOT (1) – No intervention (1)	High: 30%; Moderate: 50%; Low: 20% (Batsford scale)	The results of the RCTs showed that the use of the Suits + CT improved proximal stability, gross motor function, and gait. The other studies corroborated these findings.

Note: CT: Conventional therapy; CP: Cerebral palsy; RCT: Randomized clinical trial; QE: Quasi-experimental; GMFSC: Gross Motor Function Classification System; DEFO: Dynamic Elastomeric Fabric Orthosis; OT: Occupational therapy; NR: Not reported.

**Table 4:** Characteristics of the clinical trials.

Study	Methodological Quality *	Aims	Sample Size	Suit Groups		Comparison Groups		Measuring Instrument	Results
				Sample	Intervention	Sample	Intervention		
Emara et al., 2024 [145]	6/10	To investigate the effect of TheraTogs on foot pressure distribution, postural control, and endurance in children with CP	34	n: 17, Age: 8.57 ± 0.73 years GMFCS: I and II	Use of TheraTogs during ADLs; Parents trained and monitored weekly. Intensity: 8 to 10 h/day, for 12 weeks	n: 17 Age: 8.33 ± 0.61 years GMFCS: I and II	CT based on Bobath principles Intensity: 60 min per session, 3 times/week, for 12 weeks	Foot pressure distribution, Trunk control measurement scale, trunk position sense, 6MWT	Both groups showed significant improvements in all outcomes (*p* < 0.05).
El-Shamy and Kafy, 2021a [146]	5/10	To compare the effects of FES and TheraTogs on gait and balance in children with CP	30	n: 15, Age: 10.13 ± 1.25 years GMFCS: I and II	TheraTogs (unspecified protocol) + CT Intensity: 2 h/day, 3 times/week, for 3 months	n: 15 Age: 10.53±1.24 years GMFCS: I and II	FES in the fibular muscles + CT Intensity: 2 h/day 3x/week, for 3 months	Pro-Reflex Movement Analysis, Biodex Balance System	Both groups showed significant improvements in all outcomes. There was greater improvement in the FES group (*p* < 0.05)
El-Shamy and Kafy, 2021b [147]	5/10	Investigate the effectiveness of TheraTogs on gait pattern in children with CP	30	n: 15, Age: 10.13 ± 1.25 years GMFCS: I and II	TheraTogs (unspecified protocol) Intensity: 12 h/day.	n: 15 Age: 10.53 ± 1.24 years GMFCS: I and II	CT (stretching, strengthening and reflex inhibition). Intensity: 1h/day 3x/week, for 3 months	Pro-Reflex Movement Analysis	Both groups showed significant improvements in gait parameters. There was greater improvement in the TheraTogs group (*p* < 0.05)
El-Bagalaty and Ismaeel, 2021 [148]	4/10	Compare the use of Suit Therapy and whole-body vibration on the bone mineral density of children with CP	46	n: 23, Age: 5.13 ± 0.74 years GMFCS: NI	Use of the suit during progressive standing exercises + CT Intensity: 3 times/week, for 12 weeks	n: 23 Age: 5.0 ± 0.84 years GMFCS: NR	Whole-Body Vibration Training with Horizontal Displacements + CT Intensity: 20 min per session, 3x/week, for 12 weeks	Bone mineral density in the lumbar spine and femoral neck.	Bone mineral density improved in the whole-body vibration therapy group but not in the Suit therapy group (*p* < 0.05)

Note: CT: Conventional herapy; CP: Cerebral palsy; GMFSC: Gross Motor Function Classification System; FES: Functional Electro Stimulation; ADL: Activities of Daily Living; 6MWT: 6-Minute Walk Test; NR: Not reported. * Methodological quality assessed by the PEDro Scale.

Several studies highlight the relevance of mechanoreceptors in fascial layers for the proprioceptive capacity of fascia, with especially dense innervation in the superficial layers of the deep fascia [117,149]. Histological studies demonstrate the presence of muscle spindles and Golgi tendon organs in the deep fascia, which reinforces the proprioceptive function [149]. All of these structures are fully integrated into the fascial connective tissue and can detect any tensional variation in the fascial structure or stretching of the muscle and fascia [117]. These elements underscore the importance of fascia as a sensory system. This implies that the functional efficiency of muscle spindles is directly associated with the quality of the connective tissue present within and around the muscles [117]. Therefore, modifications in myofascial tissue have the potential to distort the information transmitted to the central nervous system. Additionally, due to its dense sensory innervation, which includes nociceptive fibers, fascia may play a significant role in pain perception. As a result, changes in myofascial tissue can impair the proper regulation of muscle tone and affect the quality of executed movements.

**Figure 3:**
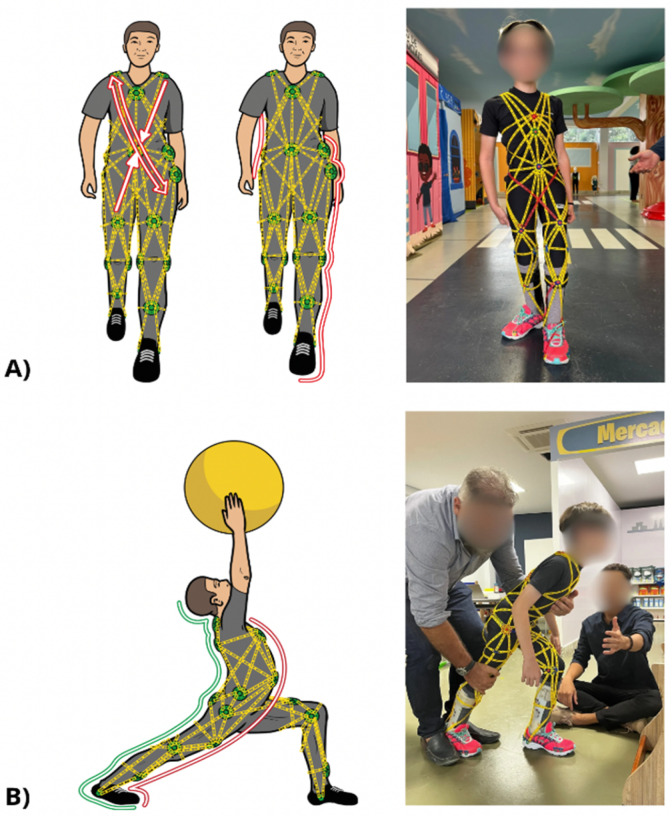
Myofascial meridians (lateral lines, spiral line, functional lines, superficial back line, and superficial front line) being stimulated by TREINI Exoflex during the execution of functional movements. This stimulation aims to allow muscles to work more efficiently, improving movement and coordination. (**A**) The lateral lines run along each side of the body. These lines provide balance between the front and back lines (see (**B**)), connecting torso and legs. They also limit excessive rotation and slow down the lateral oscillation of the body when walking. The spiral line and functional lines are also integrated in an alternating manner during walking. (**B**) The superficial back line is stimulated during body extension and the superficial front line is stretched. The energy stored through stretching is used to facilitate movements. From hyperextension onwards, all the pathways of the superficial front line are united.

Thus, the TREINI Exoflex suit was developed based on myofascial meridians, incorporating structures that simultaneously enable stabilization, postural correction, and movement [115,116]. The TREINI Exoflex was designed to replicate the transmission of tension through these myofascial tracks, allowing for the implementation of more complex motor synergies, as well as enhancing the biomechanics of fascial tissue and, consequently, the transmission of sensory information to the central nervous system (Figure 3). This facilitates the harmonious integration of different joints and body segments in complex and organized movements. Additionally, the TREINI Exoflex provides external support to the musculoskeletal system, promoting core stability, force transmission through the myofascial lines, and postural corrections in children, without restricting their movement. Since motor control and development are influenced by interaction with the environment, with posture and movement being enhanced by the suit, the TREINI Exoflex is a tool that promotes functionality, aiming for autonomy and independence during the training of the activities proposed in the intervention.

The TREINI Exoflex suit, illustrated in Figure 2 and Figure 3, is a therapeutic innovation made of lightweight, adjustable, and comfortable fabric (84% polyamide; 16% elastane). It incorporates interconnected viscoelastic strips made of a non-toxic silicone polymer, anchored at fixation points, or “nodes”, made of nylon. These strips form force-transmission pathways that distribute tension throughout the suit and, consequently, to the wearer. Made from flexible material, the TREINI Exoflex allows activities to be performed without mobility restriction, while providing effective therapeutic support. The assembly of the TREINI Exoflex is individualized, being adjusted according to the individual postural patterns of the wearer. Initially, a basic assembly is performed, from which adjustments are made to correct the different postural imbalances observed in participants. The initial adjustment of the strips is always performed gradually, respecting each child’s tolerance.

The TREINI Exoflex should not be used in isolation; its use is most effective when integrated with the performance of functional tasks. This not only enhances the therapeutic benefits of the suit, but also promotes a truly holistic approach to the treatment. The application of the TREINI Exoflex during functional tasks, especially in naturalistic environments like the “City of Tomorrow”, is crucial for facilitating postural stabilization, optimizing functional postures, and stimulating active and exploratory movements. Additionally, the TREINI Exoflex plays a fundamental role in the increase of muscle strength by transmitting force through the myofascial lines and providing resistance to muscle contraction through its strips.

The combination of stability, postural compensation, and active movement with increased muscle strength provided by the suit is achieved through the strategic distribution of stimulation along the superficial myofascial lines (anterior and posterior) and the functional lateral and spiral lines. Stimulating these myofascial lines through the training of long kinetic chains (as opposed to isolated muscle group training) can generate significant mechanical and proprioceptive benefits [141,149]. In the long term, the use of the TREINI Exoflex has the potential to enhance the transmission of accurate proprioceptive information to the central nervous system, resulting in substantial improvement in motor control. This enhancement in motor control not only optimizes the functionality and efficiency of movements, but also contributes to greater stability and postural coordination, promoting a more effective and comprehensive therapeutic approach.

The time necessary for the use of the TREINI Exoflex is estimated based on the tissue remodeling period of collagen fibers. It is estimated that approximately half of the collagen fibrils are replaced annually [140]. Extrapolating these data, in two years, about 75% of the collagen fibers would be renewed (depending on factors such as exercise, water intake, and nutrition). Therefore, a usage period of three years for the TREINI Exoflex is considered ideal for cases aiming for a complete change in the myofascial tissue. After this period, it is expected that the connective tissue will adapt and become more effective in transmitting accurate proprioceptive information to the central nervous system, contributing to improved motor control, postural stability, and efficiency in force transmission during movement execution.

## 8. TREINI Program Evaluation Strategy

There is a structured strategy for evaluating the TREINI program which involves the active participation of an interdisciplinary team. Initially, a comprehensive assessment of the needs and priorities of the child and their family is conducted. Based on this assessment, professionals carry out a detailed investigation of the factors influencing the specific demand, using validated and reliable instruments to evaluate each component of functionality and disability. Table 5 highlights the main instruments used by the TREINI program, both in the initial evaluation and in the analysis of results, which serve as essential measures of efficacy. When necessary, additional instruments are incorporated to meet the specificities of each case. The program prioritizes instruments that have been translated and culturally adapted for the Brazilian population, ensuring the relevance and applicability of the results in the local context. This approach not only reinforces the robustness of the evaluation, but also ensures that the interventions are culturally sensitive and effective.

Based on the information obtained from the evaluation, therapeutic goals are established in alignment with the needs and priorities of the child and their family. The definition of these goals follows the SMART criteria (specific, measurable, achievable, relevant, and time-bound) [150]. The goals are formulated considering the various contexts of the child and are established in collaboration with the family, addressing all domains of the ICF, including factors assessing participation, activity, and structure and function, as well as personal and environmental factors [151]. Each member of the interdisciplinary team focuses their efforts on their area of expertise, ensuring an integrated and effective approach. Reevaluations are essential for monitoring the intervention outcomes and use the same tests applied in the initial evaluation. Additionally, reevaluations help to identify new emerging skills, allowing for continuous therapeutic replanning adapted to the evolving needs of the child.

**Table 5:** Evaluation instrument.

Outcome	Instrument	Description	References
Body structure and function			
General cognitive ability	Raven’s Coloured Progressive Matrices	An assessment consisting of 36 items, used to estimate non-verbal reasoning in children.	Angelini et al., 1999 [152]
Muscle strength	Functional Strength Assessment (FSA)	Assesses the strength of the major muscle groups in children from 18 months onwards.	Jeffries et al., 2019 [153]
Range of motion	Spinal Alignment and Range of Motion Measure (SAROMM)	Provides an estimate of the overall spinal alignment and the range of motion and muscle extensibility in children with CP.	Jeffries et al., 2019 [153]
Balance	Pediatric balance scale (PBS)	A measure consisting of 14 items that assesses functional balance in the context of daily activities.	Franjoine et al., 2003 [154]
Orofacial motricity	Orofacial Myofunctional Evaluation Protocol with Scores (AMIOFE)	A structured set of procedures and criteria used to examine and document orofacial functions such as chewing, swallowing, breathing, articulation, and the posture of facial and oral muscles.	Felício et al., 2012 [155]
Hearing	Simplified Auditory Processing Assessment (ASPA)	Used to verify the efficiency with which an individual processes auditory information. It also identifies possible auditory processing disorders which may impair the ability to understand and interpret sounds.	Carvalho et al., 2019 [156]
Activity and Participation			
Goal achievement	Goal attainment scale (GAS)	Quantifies the achievement of goals set during an intervention program.	Kiresuk et al., 2014 [157]
Occupational performance	Canadian Occupational Performance Measure (COPM)	Assesses performance in daily activities in the areas of self-care, productivity, and leisure. It detects changes in performance over time and after intervention.	Law et al., 1990 [158]
Functionality	Pediatric Evaluation of Disability Inventory–Computer Adaptive Test (PEDI-CAT)	A computerized assessment tool, based on caregiver reports, that measures daily activities, mobility, social/cognitive aspects, and responsibility. It aims to identify functional delays and assess a child’s individual progress after therapeutic interventions.	Haley et al., 2012 [159]
Gross motor function	Gross Motor Function Measure (GMFM)	Measures the ability to perform activities such as lying down, rolling, sitting, crawling, standing, and walking.	Russell et al., 1989 [160]
Behavior	Child Behavior Checklist (CBCL)	Assesses behavioral and emotional problems in children and adolescents.	Bordin et al., 2013 [161]
Gait speed	10 m walk test (10MWT)	A simple assessment used to measure locomotor capacity in clinical and research settings.	Chrysagis, Skordilis, and Koutsouki, 2014 [162]
Mobility	Timed Up and Go (TUG)	Assesses gait and dynamic balance in children and adolescents.	Martín-Díaz et al., 2023 [163]
Manual dexterity	Nine-hole peg test (9HPT)	A standardized test that quantitatively assesses finger dexterity.	Poole et al., 2005 [164]
Behavior	Behavioral observation protocol (PROC)	A structured set of guidelines and procedures used to record and analyze the behavior of individuals in a specific environment.	Hage et al., 2012 [165]
Learning	School performance test (TDE)	A standardized tool used to assess the academic performance of students in various areas of knowledge.	Knijnik, Giacomoni, and Stein, 2013 [166]
Contextual factors			
Environmental	Craig Hospital Inventory of Environmental Factors (CHIEF)	Used to document the impact of environmental factors on the social participation of people with disabilities.	Furtado et al., 2014 [167]
Life history	Autobiography	A tool for self-exploration and understanding of an individual’s identity, based on their life history, with the potential to reveal patterns of behavior, beliefs, and values, as well as promote self-awareness.	Sommer, 2003 [168]
Classification systems			
Gross motor function	Gross Motor Function Classification System (GMFCS)	A five-point classification system used to describe the capacity of gross motor function in children with CP.	Palisano et al., 1997 [11]
Manual ability	Manual Ability Classification System (MACS)	A five-point classification system used to classify the manual abilities of children and adolescents with CP.	Eliasson et al., 2006 [12]
Communication	Communication Function Classification System (CFCS)	A five-point classification system used to classify the communicative function of children and adolescents with CP.	Hidecker et al., 2011 [169]
Mobility	Functional Strength Assessment (FMS)	A scale used to classify functional mobility in children and adolescents with CP. It takes into account the variety of assistive equipment that can be used.	Graham et al., 2004 [170]

## 9. Challenges and Strategies for Implementing the TREINI Program Effectively

A recent study demonstrated that the TREINI program is well-received by the families of children and adolescents [170]; however, its effective implementation requires seamless coordination among various specialists, including physiotherapists, occupational therapists, psychologists, speech therapists, and others. Achieving this level of interdisciplinary collaboration can be complex. To facilitate this process, ongoing training and scientific updates are offered to professionals working with the program. Additionally, implementing the program requires specialized facilities and technologies, such as the City of Tomorrow and the TREINI Exoflex therapeutic suit. Establishing and maintaining its infrastructure, as well as providing a trained and skilled interdisciplinary team, increases the program’s cost, from the high initial investment to ongoing operational costs, such as equipment maintenance, regular updates, and the need for consumables. The costs associated with participating in the TREINI program may limit access for those who could benefit the most. Therefore, it is necessary to promote sustainable methods within public and private health systems to ensure coverage of services like the TREINI program.

## 10. Final Considerations

The TREINI is a family-centered interdisciplinary intervention program specifically developed to support children and young people with CP and other neurodevelopmental disorders. This program has been carefully designed to aid the process of neurological reeducation and rehabilitation for children and adolescents who experience neuropsychomotor developmental delays. Based on the biopsychosocial model of health, TREINI adopts principles grounded in the best available evidence in pediatric rehabilitation, including intensive training, task-oriented training, and the use of natural learning environments. TREINI integrates intervention strategies that address impairments in all domains of functionality defined by the ICF. This approach is crucial because, while promoting activity and participation is essential, it does not replace the need to manage and prevent damage to body structures and functions. The theoretical foundation of TREINI led to the development of innovative components, such as the “City of Tomorrow” and the TREINI Exoflex therapeutic suit. The use of the TREINI Exoflex enhances postural control in children, allowing for more effective environmental exploration. This exploration is conducted in a structured, naturalistic, and active manner within the context of the City of Tomorrow, maximizing the therapeutic and educational benefits of the program.

## Figures and Tables

**Table 2:** Units of the City of Tomorrow.

Unit	Description	Theoretical–Methodological Foundation	References
Therapeutic suit (TREINI Exoflex) mounting	Spaces to store and assemble the flexible therapeutic suit (TREINI Exoflex).	Promote stability and postural correction, and improve balance, muscle strength, and quality of movements performed.	Loffi, 2019 [116] Stecco et al., 2023 [117]
Baby room	Early intervention, including Parental Guidance and Training for the stimulation of socio-emotional, communication, motor, and cognitive skills.	Maximizes child development through integrated and evidence-based strategies, fully exploring the brain plasticity and developmental potential characteristic of the early years of life.	Novak et al., 2017 [118] Hadders-Algra et al., 2017 [119]
Home	Training in activities of daily living, transfer and mobility, family literacy and numeracy, as well as socio-emotional, communication, and autobiographical skills.	Stimulates daily living skills through goal-directed and task-specific training. It uses family literacy and numeracy and autobiography as precursors for the development of cognitive and socio-emotional skills, as well as for learning mathematics and reading and writing.	Ko et al., 2020 [120] Novak and Honan, 2019 [121]
School	Activities developed through narrative grammar involving literacy and numeracy, socio-emotional skills, postural control, manual function, and functional mobility.	Oral and written activities involving words and numbers are based on cognitive–neuropsychological models of lexical processing. Motor interventions focus on improving motor ability in order to participate in school activities.	Cameron et al., 2012 [122] Alexander et al., 2024 [123]
Supermarket	Cognitive and motor planning, motor function training, shopping, socio-emotional and functional communication skills, and learning.	Understanding the cognitive script of grocery shopping allows the child to develop linguistic and conceptual skills in categorization and vocabulary, as well as numerical-arithmetic, social, and planning skills. Skills such as reaching, grasping, trunk rotation, and squatting, among other motor abilities, are also encouraged.	Santacreu, 2020 [124] Ko et al., 2020 [120]
Fitness space	Basic physical-sport skills, understanding of rules, and emotional regulation. Communication skills and social interaction related to sports practice.	The child develops basic motor skills, cardiorespiratory conditioning, and participation in group sports. Fundamental skills such as jumping, kicking, running, and throwing are trained, along with socio-emotional skills.	Sousa Junior et al., 2023 [125] Verschuren et al., 2007 [126]
Stimulus-control, controlled instability, and fixation rooms	Three stimulus-controlled rooms designed to develop skills in children with concentration difficulties. In each room, the level of support is adjusted, with a gradual increase in stimuli as the support decreases.	Systematic desensitization of stimuli and gradual fading of support for hypersensitivity to environmental stimuli and concentration training.	Hadad and Schwartz, 2019 [127] Williams et al., 2021 [128]
Social Rules Gym	Development of social and pragmatic skills.	Aims to develop communication skills, relationship building, understanding and respect for social norms, self-control and emotional regulation, as well as social problem-solving, to improve participants’ social interaction.	Vincent et al., 2017 [129]

## Supplementary Materials

The following supporting information can be downloaded at: https://www.mdpi.com/article/10.3390/children11101181/s1, Supplementary Material File S1: Search strategy.
