# Supplementary material for: Narrative Review of the Theoretical–Methodological Foundations of the TREINI Program
Source: Children (Basel). 2024 Sep 27;11(10):1181. doi: 10.3390/children11101181 (PMC11505838; doi:10.3390/children11101181)
Supplement: Supplementary file 1 [file children-11-01181-s001.zip › children-3219974-supplementary.pdf]

Supplementary Material File S1. Search strategy.

PEDro (2018 to June, 1st 2024)

1. Cerebral palsy
2. Suit therapy

Cochrane (2018 to June, 1st 2024)

1. Cerebral palsy
2. "Cerebral Pals*"
3. AND/ 1-2
4. Suit
5. Suit therapy
6. Garment
7. Dynamic elastomeric fabric orthos*
8. Adeli
9. TheraSuit
10. Theratog
11. "pedia suit"
12. "thera suit"
13. "adeli suit"
14. "modified suit"
15. "neuro suit"
16. "penguin suit"
17. "bungy suit"
18. Or/ 3-16
19. 3 AND 18

PUBMED (2018 to June, 1st 2024)

20. Cerebral palsy
21. "Cerebral Pals*"
22. AND/ 1-2
23. Suit
24. Suit therapy
25. Garment
26. Dynamic elastomeric fabric orthos*
27. Adeli
28. TheraSuit
29. Theratog
30. "pedia suit"
31. "thera suit"
32. "adeli suit"
33. "modified suit"
34. "neuro suit"

35. “penguin suit”
36. “bungy suit”
37. Or/ 3-16
38. 3 AND 18

Scopus (2018 to June, 1st 2024)

1. Cerebral palsy
2. “Cerebral Pals*”
3. AND/ 1-2
4. Suit
5. Suit therapy
6. Garment
7. Dynamic elastomeric fabric orthos*
8. Adeli
9. TheraSuit
10. Theratog
11. “pedia suit”
12. “thera suit”
13. “adeli suit”
14. “modified suit”
15. “neuro suit”
16. “penguin suit”
17. “bungy suit”
18. Or/ 3-16
19. 3 AND 18

Web of Science (2018 to June, 1st 2024)

1. Cerebral palsy
2. “Cerebral Pals*”
3. AND/ 1-2
4. Suit
5. Suit therapy
6. Garment
7. Dynamic elastomeric fabric orthos*
8. Adeli
9. TheraSuit
10. Theratog
11. “pedia suit”
12. “thera suit”
13. “adeli suit”
14. “modified suit”
15. “neuro suit”
16. “penguin suit”
17. “bungy suit”
18. Or/ 3-16
19. 3 AND 18
